# Numerical Study of Opto-Fluidic Ring Resonators for Biosensor Applications

**DOI:** 10.3390/s121014144

**Published:** 2012-10-22

**Authors:** Han Keun Cho, Jinwoo Han

**Affiliations:** 1 Department of Biosystems Engineering, Chungbuk National University, Cheongju 361-763, Korea; E-Mail: hkcho@cbu.ac.kr; 2 Department of Physics, Daegu University, Gyungsan 712-714, Korea

**Keywords:** ring resonator, whispering gallery mode, waveguide

## Abstract

The opto-fluidic ring resonator (OFRR) biosensor is numerically characterized in whispering gallery mode (WGM). The ring resonator includes a ring, a waveguide and a gap separating the ring and the waveguide. Dependence of the resonance characteristics on the resonator size parameters such as the ring diameter, the ring thickness, the waveguide width, and the gap width between the ring and the waveguide are investigated. For this purpose, we use the finite element method with COMSOL Multiphysics software to solve the Maxwell's equations. The resonance frequencies, the free spectral ranges (FSR), the full width at half-maximum (FWHM), finesse (F), and quality factor of the resonances (Q) are examined. The resonant frequencies are dominantly affected by the resonator diameter while the gap width, the ring thickness and the waveguide width have negligible effects on the resonant frequencies. FWHM, the quality factor Q and the finesse F are most strongly affected by the gap width and moderately influenced by the ring diameter, the waveguide width and the ring thickness. In addition, our simulation demonstrates that there is an optimum range of the waveguide width for a given ring resonator and this value is between ∼2.25 μm and ∼2.75 μm in our case.

## 1. Introduction

**Figure 1.**
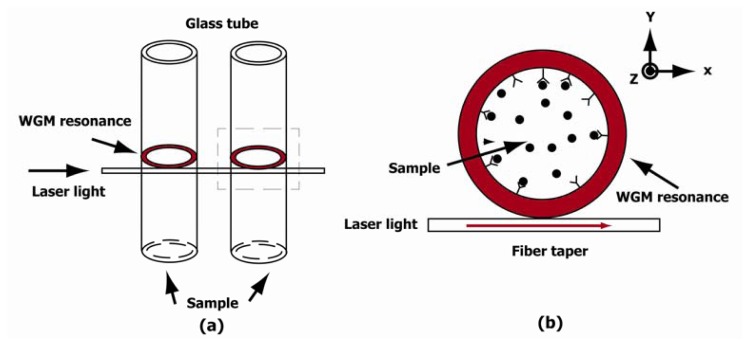
The schematic of (**a**) the multi-channel OFRR and (**b**) a section view.

The opto-fluidic ring resonator sensing platform has recently emerged as a new solution for highly sensitive detection of biological and chemical analytes [1–3]. Here, an analyte means a substance or chemical constituent that is of interest in an analytical procedure. The OFRR, which is a glass capillary-based cavity, integrates microfluidics with photonics. In a ring resonator, light propagates in the whispering gallery mode (WGM), which allows for a great miniaturization of the sensors while maintaining a longer effective interaction period. The capillary nature of the ring resonator also enables the convenient sample delivery [4,5]. The 2-dimensional array arrangement enables high efficiency detection and multiple-analyte detection, as shown in Figure 1(a). The OFRR can achieve a greatly improved detection limit, lower sample consumption, and larger integration density than possible with traditional optical sensors [6].

A high quality OFRR configuration design is essential to optimize the sensitivity of specific biomolecule detection. However, because the experimental approach to this task is expensive and time-consuming, theoretical models have been utilized to describe the WGMs of the OFRR using Mie theory by considering a three-layer radial structure. Using this model, the radial distribution of the WGMs electrical field is derived and the resonant wavelength can be obtained numerically by matching the boundary conditions. Furthermore, the WGM spectral position can be obtained as a function of wall thickness, the resonator size, operating wavelength, *etc.*, which allow us to calculate the sensitivity to refractive index change and to optimize the OFRR design.

The binding of analytes to the resonator inner surface results in a modification of the effective refractive index and the ring thickness experienced by the WGM, leading to change in the WGM spectral position. A number of analytical studies have been conducted to understand the evanescent coupling for various resonator designs [7–13]. Though these studies have provided valuable information on the resonance characteristics of resonators and helpful guides for experimental works, we can get a more realistic picture of the resonator system through a numerical simulation. The Mie theory is, for example, not enough to explain the coupling of the evanescent fields in the nanoscale gap. As a matter of fact, the electromagnetic (EM) field in the resonator has a high sensitivity to the gap through which tunneling of photons occurs. Accordingly, it is highly desirable to model the EM field of the whole WGM system and investigate numerical characterization which is essential in developing an efficient and optimized structure of an OFRR sensor.

In this study, the finite element method is used for solving the Helmholtz equation of electromagnetic waves reduced from Maxwell's equations. The commercial COMSOL Multiphysics package (version 4.1) is applied to perform a finite element analysis in the OFRR simulation. We mainly investigate the resonance characteristics associated with the ring diameter, the ring thickness, the gap width, and the waveguide width. We also discuss in detail the effects of these parameters.

## 2. Theory

The sketch of a conceptual OFRR is shown in Figure 1(b). The electromagnetic field in the WGM of the OFRR can be described by time-dependent Maxwell's equations. In source-free non-conducting media, the wave equation can be written as:

(1) $$\nabla^{2}\vec{E}-\frac{n^{2}}{c^{2}}\frac{\partial^{2}\vec{E}}{\partial t^{2}}=0$$

where n is the refractive index of the media and *c* is the speed of light in free space. For time-harmonic fields such as *E⃗*(*r⃗*, *t*) = *E⃗*(*r⃗*)*e*^−^*^iωt^*, Equation (1) is rewritten as:

(2) $$\nabla^{2}\vec{E}+\frac{n^{2}\omega^{2}}{c^{2}}\vec{E}=0$$

When the resonance ring is placed in the (x, y) plane as shown in Figure 1(b) and the electric field is polarized along the z direction, the transverse electric (TE) field is given by *E⃗*(*r⃗*) = *E*(*x*,*y)z⃗*. In this case, the Equation (2) becomes a scalar Helmholtz equation:

(3) $$\nabla^{2}\vec{E}+n^{2}k_{0}^{2}\vec{E}=0$$

where k_o_ is the free-space wave number.

For simplicity, the resonance ring is placed on the same plane as the waveguide, as shown in Figure 1(b). Thus, WGM can be treated in a two-dimensional model. For outside boundaries, the scattering boundary condition is applied with initial amplitude of *E*_0_ = 0 For laser excitation source, the scattering boundary condition is also used with input amplitude of *E*_0_ = 1*V*/*m* The scattering boundary condition is commonly used to specify a boundary which is transparent for a scattered wave and for an incoming plane wave.

## 3. Simulation

The finite difference method (FDM) has been commonly used for numerical studies in electromagnetics. However, the finite element method (FEM) has an advantage in dealing with irregular configurations or system analysis. In this study, the FEM is used to solve the Helmholtz equation. COMSOL Multiphysics with RF module (version 4.1, COMSOL Inc., Burlington, MA, USA) is employed for numerical analysis and post processing.

**Figure 2.**
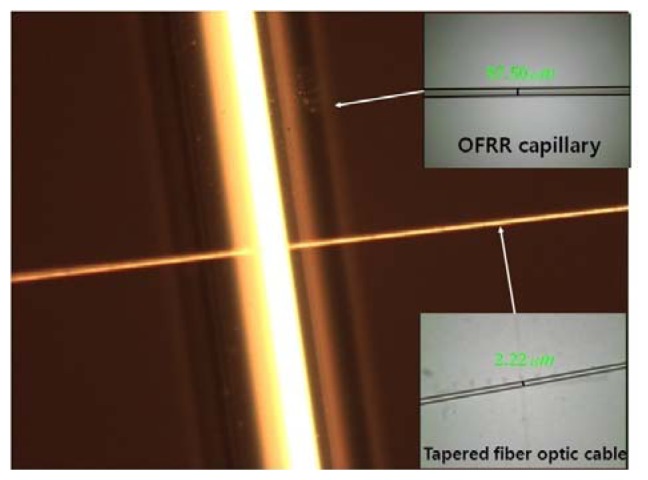
A photograph of a fabricated OFRR.

A glass capillary and an optical fiber are used as the ring resonator and the waveguide, respectively. Figure 2 shows a photograph of a fabricated OFRR system under construction. A simulation domain is typically a 140 μm × 140 μm rectangular area. A ring resonator is centrally located and a waveguide is positioned below the resonator. Separation distance between the resonator and the waveguide can be adjusted by varying a small gap. The waveguide length is the same as the width of the simulation domain. The outer diameter and the thickness of the ring resonator are 100 μm and 3 μm respectively, and the width of the waveguide is 2 μm. The surrounding material for the ring and the waveguide is assumed as air and the material inside the ring is regarded as water. The refractive indices of the resonator and the waveguide are set to be 1.52 (glass) and 1.4682 (SiO_2_), respectively. The refractive indices for air and water are 1.0 and 1.333, respectively. A tunable laser beam for resonance excitation is directed straight from the left end of the waveguide. The frequency of the incident laser ranges between 192.31 THz (1,560 nm) and 196.80 THz (1,530 nm). It is noted that the wavelength *λ* and frequency *f* are the values in free space, unless otherwise specified.

A mesh is a discretization of the geometry model into small simple shapes, and in this work the computational domain is meshed by triangle elements. The normal mesh size predefined in COMSOL is selected. All domains are meshed in two steps; the free triangular step followed by the refine step. The free triangular step is taken once to all domains. The refine step is taken thrice for the domains of the resonator and the waveguide. For the remaining domains of air and the sample, two refine steps are taken. The ratio of average mesh size in the domains for air and the sample to that in the domains for the resonator and the waveguide is about 58.3 to 1. Though the computational resolution of the laser wavelength is basically 0.5 nm, a 0.01 nm resolution is chosen in the vicinity of the resonance frequency for accuracy. The detailed simulation procedure using the COMSOL Multiphysics package is described elsewhere [14].

The distributions for electric fields and radiation energy density were examined under two WGM resonances (1st and 2nd order) and off resonance, respectively. We compared the experimental results for three operating conditions. The diameter and the thickness of the resonator ring were 100 μm and 3 μm, respectively. The waveguide thickness was 2 μm and the gap width between the resonator and the waveguide was 0 nm.

In addition, we conducted four parametric studies. In order to find the effects of each parameter, we varied only one parameter at a time with other three parameters fixed. The diameter of the resonator (d) was varied between 80 and 120 μm with 10 μm step. The thickness of resonator (t) was controlled between 2.5 and 4.0 μm with 0.5 μm step. The width of the waveguide (w) was changed between 1.5 and 3.25 μm with varied step size. The gap size between the resonator and the waveguide (g) was varied between 0 and 300 nm with 100 nm step. For accuracy, we obtained four sets of scattering spectra for each parameter in the frequency range of 192 THz to 196 THz. Then, we extracted the resonance characteristics from the scattering spectra to investigate the resonator configuration effects. Our study was mainly focused on the resonance characteristics such as the resonant frequency, the full width at half-maximum of the resonant frequency band, the quality factor defined by the resonant frequency divided by FWHM, the free spectral range, and the finesse of the resonant mode defined by FSR divided by FWHM.

## 4. Results and Discussion

### 4.1. Electric Fields and Radiation Energy Distribution

**Figure 3.**
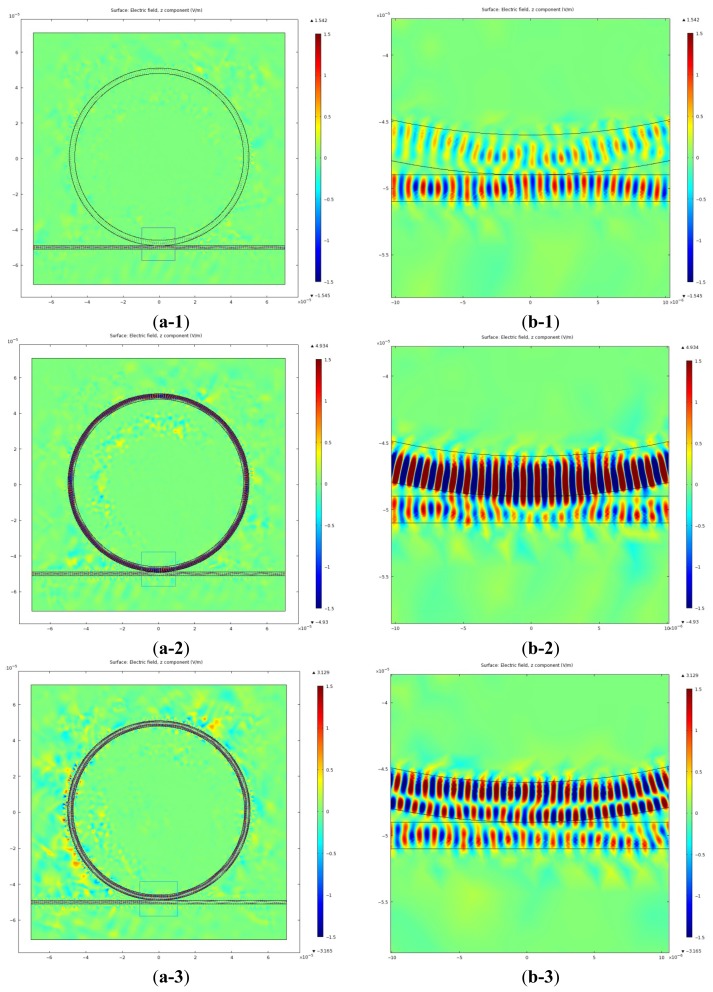
Electric fields distributions for the ring-waveguide coupling part (d = 100 μm, t = 3 μm, w = 2 μm, g = 0 μm); (**a**) the regular plots and (**b**) the magnified plots. (Off-resonance (**a-1**,**b-1**); first-order resonance (**a-2**,**b-2**); second-order resonance (**a-3**,**b-3**)).

The first and second order resonances are found at *λ* = 1,550.55 nm (193.346 THz) and at *λ* = 1,552.80 nm (193.066 THz) respectively. The off-resonance is chosen at *λ* = 1,554.45 nm (192.861 THz). Figure 3 shows the distributions for electric fields and radiation energy density for the three frequencies. In Figure 3, the first row shows regular size plots and the second row shows the magnified plots for the coupled region of the ring and the waveguide.

In Figure 3, it is clearly seen that a weak EM field exists in the resonator, even under the off-resonance condition. That is because an evanescent field couples from the waveguide to the resonator when the gap width is comparable to or less than one optical wavelength of the field. Accordingly, under the off-resonance condition, the EM field is mainly confined in the waveguide and its strength in the resonator is very weak. However, on the first-order resonance, a strong EM field is formed inside the resonator close to the peripheral boundary. The EM field in the ring becomes much stronger than that in the waveguide. More interestingly, when the second-order resonance occurs, two different resonance rings composed of strong EM fields are built inside the ring. These two low-order resonances, as shown in Section 4.2, are the radial solutions of the Equation (2), and many high-order resonances, not discussed here, could also exist when the boundary conditions are satisfied. In the case of the second-order resonance, the EM field is observed to be about 1.5 times stronger in the inner ring than in the outer ring. The ratio of EM energy stored in the ring to the energy stored in the wave guide goes up to 34.13 and 10.84 for the first and the second order resonance, respectively. This ratio drops to 0.75 in the case of the off-resonance. In general, the high-order resonances are more preferred for the sensor application because they build stronger evanescent EM fields inside the inner ring where interaction with the analytes occurs. However, we selected the first-order resonance for this numerical study for two reasons. First, study on the first-order resonance is important for comparison with the high-order resonance studies in progress. Second, the EM energy confined in the ring is much higher for the first-order resonance than for the second-order resonance, as seen in Section 4.2. Because the enhanced WGM wave circulates along the ring surface and interacts repeatedly with the analytes, it is emerging as a promising sensor device for detecting nanoparticles and viruses as well as chemical/biological molecules in the future.

### 4.2. Effect of the Resonator Diameter on Scattering Spectra and Resonance Characteristics

**Figure 4.**
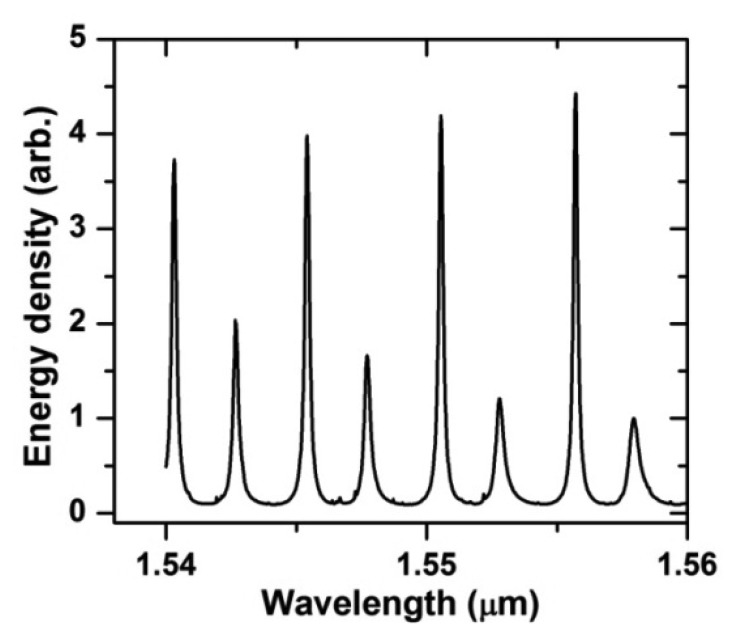
Resonance spectra of the ring for the OFRR (d = 100 μm, t = 3 μm, w = 2 μm, g = 0 nm). The large peaks are for the first-order resonances while the small peaks are for the second-order resonances.

**Figure 5.**
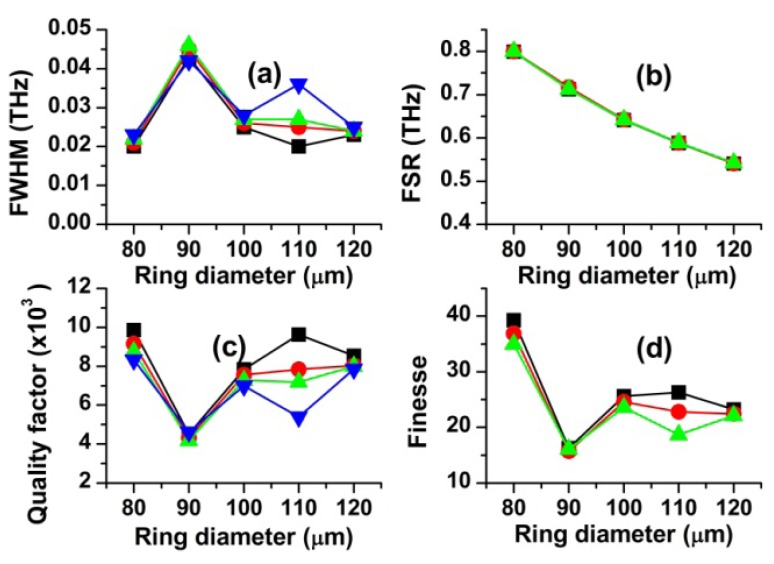
Effects of the ring diameter on the resonance characteristics (t = 3 μm, w = 2 μm, g = 0 μm). Displayed four data sets are obtained from adjacent resonance peaks, respectively.

Figure 4 shows the EM energy density of the ring (J/m) as a function of the wavelength for the OFRR. The ring diameter, the ring thickness, the waveguide width and the gap width were 100 μm, 3 μm, 2 μm, 0 nm, respectively. In this study, we obtained four sets of adjacent first-order resonance data for each resonator diameter and plotted four resonance characteristics corresponding to these resonance data using different symbols, as shown in Figure 5. We conducted the same procedure for other three parameters unless otherwise specified.

In the actual simulation process, we solve Maxwell's equations for a given resonator structure and material properties. Therefore, our simulation results naturally include the power loss in the ring as well as the coupling power loss between the waveguide and the ring. The resonance characteristics determined from this simulation are realistic, and therefore it should be noted that the quality factor Q discussed in this work is the total Q, defined by 1/Q = 1/Q_external_ + 1/Q_internal_.

In Figure 5, it is interesting to note that FSR monotonically decreases with increasing diameter of the ring while FWHM, quality factor and finesse depend irregularly on the resonator diameter. These results indicate that only FSR is strongly affected by the resonator size. Furthermore, large finesse values make the WGM resonators excellent candidates for detection of various analytes using the spectroscopy method. The decreasing dependence of FSR on the ring diameter can be explained by the resonant mode of light orbiting in the ring. When a *m-th* resonant mode is developed in the ring by the total internal reflection, frequency of light is given by:

(4) $$f(m,l)=\frac{mc}{2\text{nlr}\sin(\pi /l)}$$

where *c* is the speed of light in free space, *n* is refractive index of the ring, *m* is the mode number, and r is the outer radius of the ring. *l* is the number of sides of a *l*-sided polygon formed by light ray traveling around the outer boundary of the ring by the total internal reflection. When *l* is large as in our case, the Equation (4) can be approximated as *f* ≅ *mc*/*2πnr*, and *FSR* = *c/2πnr* for Δ*m* = 1. Therefore, FSR is inversely proportional to *r*, as observed in Figure 5(b).

### 4.3. Effect of the Resonator Thickness on Scattering Spectra and Resonance Characteristics

**Figure 6.**
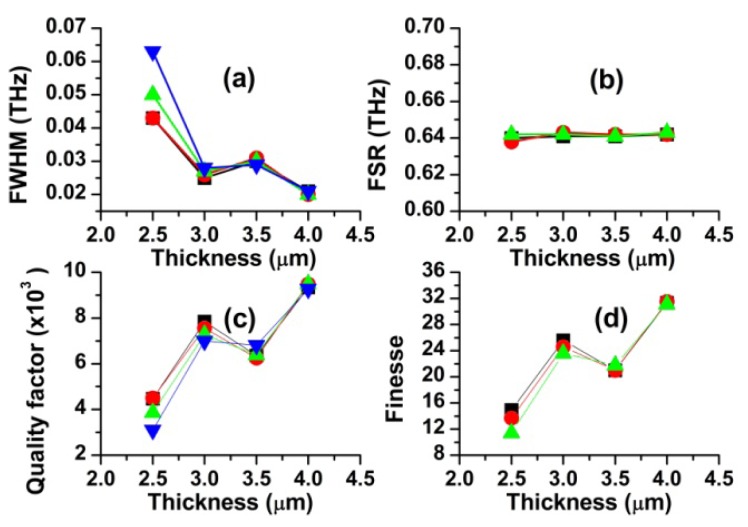
Effects of the ring thickness on the resonance characteristics (d = 100 μm, w = 2 μm, g = 0 μm). Displayed four data sets are obtained from adjacent resonance peaks, respectively.

In order to study dependence of resonance parameters on ring thickness, we calculated the scattering spectra for four different OFRR thicknesses (t = 2.5, 3.0, 3.5 and 4.0 μm). The ring diameter, the gap width and the waveguide width were 100 μm, 0 nm and 2 μm, respectively. Four first-order resonant frequencies (modes) were found for each of the resonator thickness in the frequency range of 192–195 THz. The resonant characteristics obtained from the scattering spectra (not shown) are displayed in Figure 6. It is clearly seen from Figure 6 that the ring thickness negligibly affects FSR, but it significantly does FWHM, Q and F. In particular, it is interesting to note FWHM increases with decreasing ring thickness. As the ring thickness decreases, electromagnetic energy confined in the ring more easily leaks out via the evanescent field, as seen in Figure 3. Quality factor is defined by *Q* = *2πE_s_/E_d_* = *f/FWHM*. Here, *E_s_* is the stored energy, *E_d_* is the dissipated energy per cycle, and *f* is the resonance frequency. Therefore, the increased dissipation energy through the evanescent field naturally brings about increase in FWHM and decrease in Q and F. Negligible change in FSR with varying the ring thickness can be explained by Equation (4). As the ring thickness changes, only the inner radius of the ring varies while the outer radius of the ring remains fixed. Because the light wave is travelling around the outer boundary of the ring in the whispering gallery mode, resonance frequency f and FSR are little affected.

### 4.4. Effects of the Waveguide Width on Scattering Spectra and Resonance Characteristics

**Figure 7.**
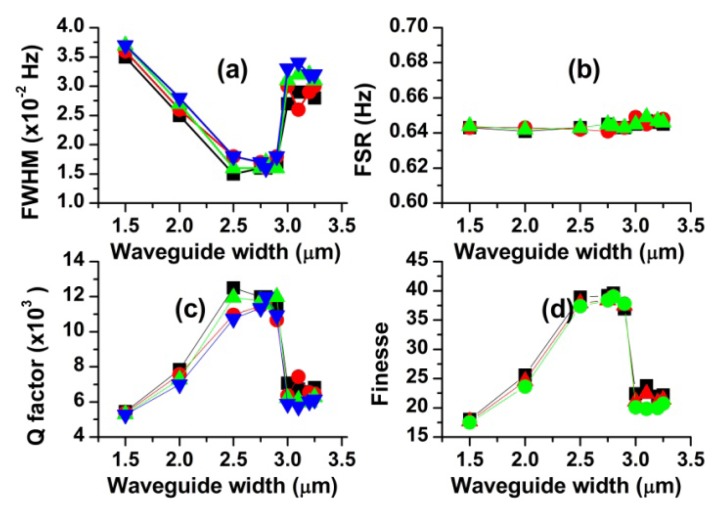
Effects of the waveguide width on the resonance characteristics (d = 100 μm, t = 3 μm, g = 0 μm). Displayed four data sets are obtained from adjacent resonance peaks, respectively.

In order to understand how the resonance parameters are affected by the waveguide width, we simulated the scattering spectra for ten different waveguide widths (w = 1.5–3.25 μm). The ring diameter, the gap width and the ring thickness were 100 μm, 0 nm and 3 μm, respectively. The resonant characteristics obtained from the scattering spectra (not shown) are plotted in Figure 7. As shown in Figure 7, FWHM, F and Q markedly changes with increasing the waveguide width whereas FSR insignificantly varies. Because the size of the resonance ring has not changed at all, negligible change in the resonance wavelength is expected from Equation (4). To be more specific, as the waveguide width increases, FWHM first rapidly decreases, then reach a minimum in the range of 2.5–2.9 μm, and afterwards sharply increases at 3.0 μm, eventually approaching a value. Both Q and F show similar behaviors, but in the opposite direction.

**Figure 8.**
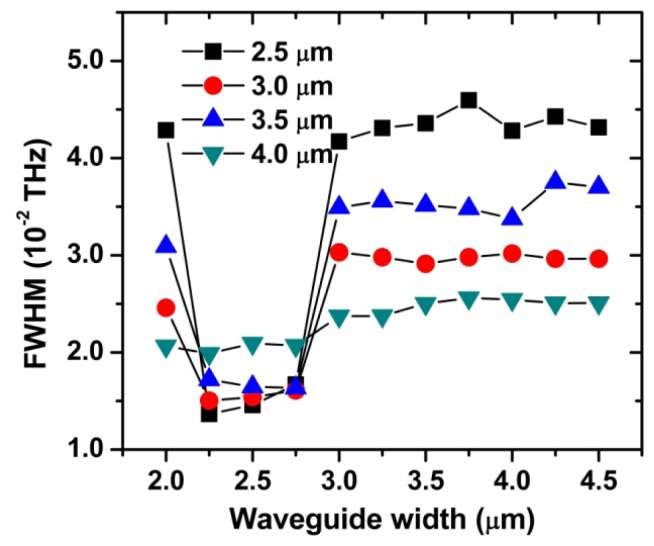
Effect of the waveguide width on FWHM (d = 100 μm, g = 0 μm).

The sharp jumps of Q, F and FWHM near 3.0 μm are quite surprising. In order to investigate this interesting behavior in more detail, we extended our study for four different ring thicknesses (t = 2.5, 3.0, 3.5, 4.0 μm). The simulated FWHM values plotted in Figure 8, which are similar to those in Figure 7, show several features to discuss here. First, there is an optimum range of the waveguide width (w = 2.25–2.75 μm), regardless of the ring thickness. Second, FWHM rapidly changes at w ≈ 2.25 μm and 3.0 μm. In particular, these changes are most pronounced for the ring thickness 2.5 μm and become smaller as the ring thickness is increased (except for t = 3 μm case). Third, FWHM is hardly affected by the waveguide width in the range of w = 3.0–4.5 μm.

With increasing waveguide width, the electric field in the waveguide extends less into the ring resonator and coupling strength between them decreases. The decreased coupling strength causes Q and F to increase and FWHM to decrease in the range of w = 1.5–2.5 μm, as seen in Figure 7. FWHM in Figure 8 shows a similar behavior in the range of w = 2.0–2.25 μm. In this region, the thinner the ring thickness is, the more FWHM seems to be affected by the waveguide width. Because further increase in the waveguide width has no effect on additionally decreasing the coupling strength and FWHM, our ring resonator can operate optimally in the range of w = 2.25–2.75 μm.

The sharp jump of FWHM near 3.0 μm, as displayed in Figure 8, needs another physical explanation. We can infer, from the smooth change in FWHM for the resonator with t = 4 μm, that this jump is strongly associated with the small size of the ring thickness. One probable explanation is that the coupling phase-mismatch considerably decreases for some reason when the waveguide width becomes comparable to 3.0 μm. Decreased phase-mismatch increases the coupling efficiency and therefore increases the FWHM values [7]. As shown in Figure 8, FWHM is nearly constant in the range of w = 3.0–4.5 μm. It indicates that the critical factor, in this region, is the ring thickness rather than the waveguide width. Furthermore, it demonstrates that the resonator with a large ring thickness is more suitable for the high Q device.

### 4.5. Effect of the Gap Width on Scattering Spectra and Resonance Characteristics

**Figure 9.**
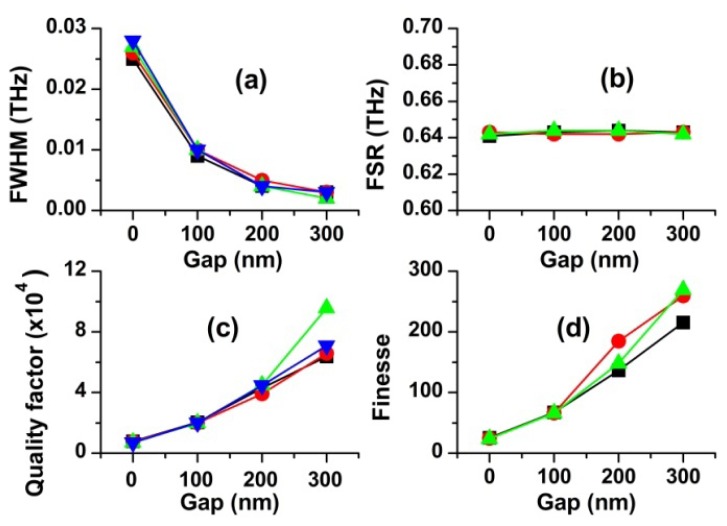
Effects of the gap width on the resonance characteristics (d = 100 μm, t = 3 μm, w = 2 μm). Displayed four data sets are obtained from adjacent resonance peaks, respectively.

Finally, we investigated how the resonance parameters are influenced by the gap width. For this purpose, we simulated the scattering spectra for four different gap widths (g = 0, 100, 200 and 300 nm). The ring diameter, the waveguide width and the ring thickness were 100 μm, 2 μm and 3 μm, respectively. Four first-order resonant frequencies (modes) are found for each of the gap width in the frequency range between 192 THz and 195 THz. The resonant characteristics obtained from the scattering spectra (not shown) are plotted in Figure 9.

It is clearly seen in Figure 9 that the effect of the gap width on the resonant frequencies and their intervals (FSR) is negligible. In contrast, FWHM, F and Q are strongly affected by the gap width. For example, with the increase of the gap width from 0 to 300 nm, FWHM decreases by over an order of magnitude and Q and F increase by the same factor. Our results are consistent with the simulated works reported for a micro-disk resonator by a research group [15].

**Figure 10.**
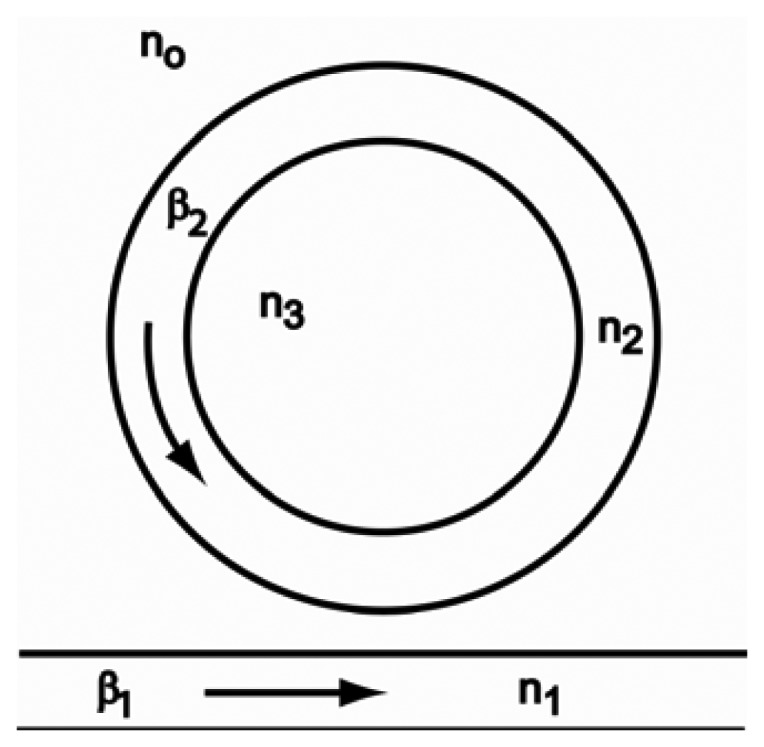
Schematic diagram of the ring resonator coupled to a waveguide.

These interesting results can be qualitatively explained by the coupled-mode theory (CMT) [16]. Figure 10 shows a schematic diagram of the ring resonator coupled to a waveguide. In Figure 10, *β_1_* and *β_2_* are the propagation constants in the waveguide and in the ring, respectively. n_0_, n_1_, n_2_ and n_3_ are the refractive indices of air, the wave guide, the ring and water, respectively.

**Figure 11.**
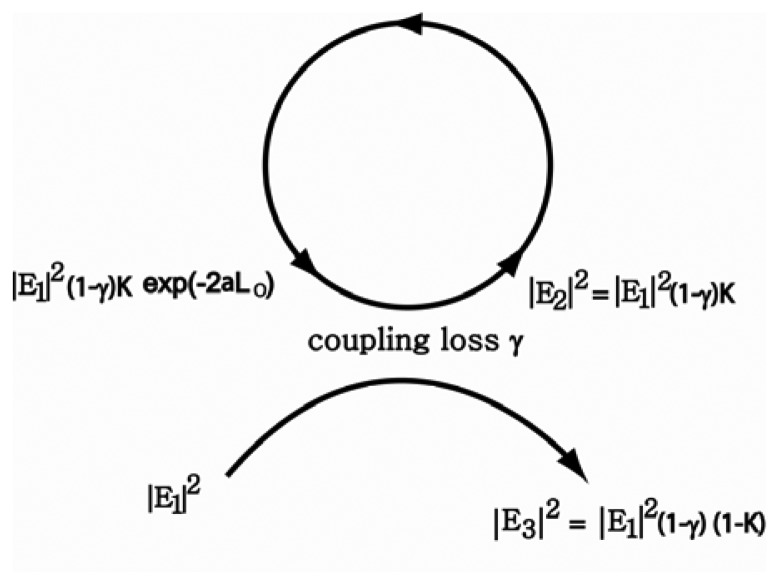
Model of wave coupling.

This resonator can be modeled by a coupled system with several physical parameters, as depicted in Figure 11. In this model, we take two sources of loss into account. γ is the coupling power loss factor between the waveguide and the ring, and α is the amplitude loss coefficient of the electric field in the ring. In addition the power splitting ratio *K* is introduced as illustrated schematically in Figure 11. Here, K is defined as the ratio of the power coupled to the ring resonator to the total power input into the waveguide and it mainly depends on the geometrical shape of the coupled system. A lengthy calculation based on this model gives:

(5) $$\frac{P_{2}}{P_{2}^{m}}=\frac{KK_{0}}{1+(1-K)(1-K_{0})-2\sqrt{(1-K)(1-K_{0})\cos\varphi }}$$

In Equation (5)*P*_2_ = |*E*_2_|^2^/|*E*_1_|^2^, $P_{2}^{m}=(1-\gamma )/K_{0}$, and *K*_0_ = 1−(1 − *γ*)*e*^−2^*^αL^*^0^. $P_{2}^{m}$ is the maximum value of P_2_ on resonance and K_0_ is the power splitting ratio on resonance. K_0_ is an intrinsic resonance parameter related to the power loss of the system. Therefore, it can be shown that $0\leq P_{2}^{m}\leq 1$. The phase change *φ* is defined by *φ* = *β*_2_*L*_0_ + *β_av_L_c_*. *φ* relies highly on the refractive index and the wavelength of light in the waveguide and in the ring. *β_av_* is the effective propagation constant in the coupling region. *L_c_* is the length of the coupling region and *L*_o_ is the length of ring excluding the coupling region. They are related to each other by *L*_0_ = 2*πr* − *L_c_*. On resonance, the power coupled to the ring must exactly offset the power losses through the coupler and from circulation around the ring. Furthermore, phase change is given by *φ* = 2*nπ* (n = integer) on resonance. For a given geometrical configuration with a fixed *K*, the power in the ring is determined by the wavelength of light through the phase change *φ*. By plotting Equation (5) as a function of *K* and *φ*, it was demonstrated that the resonance curve becomes narrower as *K*_0_ is smaller [16]. For example, if no power is lost while light is travelling around the ring (*α* = 0), *K*_0_ = *γ*. In this case, if we adjust *K* to *γ*, we can get the narrowest resonance curve. Because resonance occurs at *K* = *K*_0_ and K generally decreases nearly exponentially with increasing the gap width, FWHM is expected to decrease with increasing gap width. Our results are in good agreement with CMT, as observed in Figure 9. As *K* approaches *K_0_*, decrease in FWHM slows down. It is obvious that increases in Q and F with increasing gap width result from the decreased FWHM. However, it should be noted that a large gap width is unfavorable to storing energy in the ring, and therefore a trade-off between them is necessary.

## 5. Conclusions

WGM resonators with a capillary ring-waveguide coupling arrangement were numerically characterized by FEM. By solving Maxwell's equations, the electric fields and radiation energy distributions in the rings were determined. As WGM resonance occurs, a very shining loop with a strong electric field and high radiation intensity exists inside the periphery of the ring resonator under first-order resonance. There are two glittering loops inside the ring under the second-order resonance and the light intensity of the inner loop is higher than that of the outer loop. Thus, the second order resonances may be preferred in sensing applications because interesting interactions with analytes occur mainly in the vicinity of the inner boundary of the ring through the evanescent field.

The WGM resonant frequencies are predominantly determined by the resonator diameter. Contrastingly, the gap width, the ring thickness and the waveguide width have negligible effects on the resonant frequencies.

FWHM, the quality factor Q and the finesse F are most substantially affected by the gap width and moderately influenced by the waveguide width and the ring thickness. For example, with increasing the gap width from 0 to 300 nm, Q and F increase tenfold while FWHM decreases by one tenth. Contrastingly, FWHM, Q and F vary by a factor of 2 as the ring diameter, the ring thickness and the waveguide width change.

In addition, our simulation demonstrates that there is an optimum range of the waveguide width for a given ring resonator and this value is between ∼2.25 μm and ∼2.75 μm in our case.
